# The Impact of Essential Trace Elements on Ovarian Response and Reproductive Outcomes following Single Euploid Embryo Transfer

**DOI:** 10.3390/ijms241310968

**Published:** 2023-06-30

**Authors:** Roberto Gonzalez-Martin, Andrea Palomar, Alicia Quiñonero, Nuria Pellicer, Rocio Fernandez-Saavedra, Estefania Conde-Vilda, Alberto J. Quejido, Christine Whitehead, Richard T. Scott, Francisco Dominguez

**Affiliations:** 1 IVIRMA Global Research Alliance, IVI Foundation, Instituto de Investigación Sanitaria La Fe (IIS La Fe), 46026 Valencia, Spain; roberto.gonzalez@ivirma.com (R.G.-M.); andrea.palomar@ivirma.com (A.P.); alicia.quinonero@ivirma.com (A.Q.); nuria.pellicer@ivirma.com (N.P.); 2 Unit of Mass Spectrometry and Geochemical Applications, Chemistry Division, Department of Technology, CIEMAT, 28040 Madrid, Spain; 3 IVIRMA Global Research Alliance, IVI-RMA New Jersey, Basking Ridge, New Jersey, NJ 07920, USA; 4 Sidney Kimmel College of Medicine, Thomas Jefferson University, Philadelphia, PA 19044, USA

**Keywords:** essential trace elements, biofluids, ovarian response, IVF outcomes, ICP-MS

## Abstract

Essential trace elements are required in extremely small amounts and obtained through diet. This research focuses on detecting major trace elements in different biofluids of sixty women undergoing ICSI with PGT-A and SET/FET at IVI-RMA, New Jersey, and assessing their impact on their IVF outcomes. Urine, plasma, and follicular fluid samples were collected on the vaginal oocyte retrieval day to measure the concentrations of eight essential trace elements (copper, zinc, molybdenum, lithium, selenium, manganese, chromium, and iron) using ICP-MS. After analysis, ovarian response and preimplantation outcomes had significant positive associations with both copper alone and the copper/zinc ratio in the follicular fluid and plasma, in addition to plasma manganese. Alternatively, elevated follicular fluid lithium concentrations were significantly associated with poor preimplantation outcomes while the urinary molybdenum concentration was significantly associated with a lower probability of implantation, clinical pregnancy, and live birth. Urinary lithium and chromium concentrations were significantly associated with a lower probability of achieving a live birth. Our results suggest that the essential trace elements present in follicular fluid, plasma, and urine of women are directly associated with their reproductive outcomes, with copper and manganese exerting positive effects and lithium and molybdenum exerting negative effects.

## 1. Introduction

In industrialized countries, approximately 16% of women of reproductive age undergo assisted reproduction treatments (ARTs) [1,2]. Indeed, subfertility is estimated to affect more than fifty million couples worldwide [1,2], with conception difficulties mainly caused by poor gamete and embryo quality and/or implantation failure [1]. Accompanying the increased awareness that human fertility is affected by genetic, sociodemographic, environmental, and nutritional factors [3,4], the latter two are gaining relevance in subfertility management as they can be partially controlled for with preventive lifestyle changes and/or therapeutic approaches [3].

Among the nutritional factors that can compromise fertility, dietary trace elements (e.g., copper [Cu], zinc [Zn], molybdenum [Mo], lithium, selenium, manganese, chromium, and iron) are of particular importance because they are indispensable for normal physiological function, despite their concentrations representing <0.6% of the body burden [4,5]. These trace elements provide structural stability for other essential molecules, in addition to enzymatic activity (as cofactors or catalysts), contributing to protein synthesis and regulation of gene expression [4,5]. Their involvement in a broad range of biological processes, particularly cellular metabolism and antioxidant defenses, makes them especially relevant for the acquisition of oocyte competence [6]. Essential trace element homeostasis is tightly regulated and their deficiencies can impair reproductive success [4,7,8,9]. Several studies suggested that subfertile women undergoing IVF had lower concentrations of essential trace elements [8,9,10] and supplementation may improve their outcomes [7,9]. Conversely, out-of-range levels of any trace elements can adversely affect reproduction and other organic functions [4,5].

Few studies have previously evaluated the impact of female essential trace elements in different biofluids on IVF outcomes, with inconsistent findings [11,12,13]. Further, these studies have primarily focused on zinc, with limited data to support the roles of other essential trace elements on in vitro fertilization (IVF) outcomes [11,12,13]. Thus, this study aimed to evaluate the impact of multiple essential trace elements, with IVF outcomes, using the concentrations found in the follicular fluid (FF), plasma, and urine of women undergoing intracytoplasmic sperm injection (ICSI) with preimplantation genetic testing for aneuploidies (PGT-A) and single frozen embryo transfer (SET/FET).

## 2. Results

### 2.1. Baseline Characteristics

Among the sixty participants, the median age and BMI were 33.40 years (IQR: 31.37, 36.50) and 23.87 kg/m^2^ (IQR: 21.57, 26.30), respectively; 71.7% were Caucasian and 81.7% had never smoked (Table 1).

Participants’ median anti-Müllerian hormone (AMH) concentration was 3.60 ng/mL (IQR: 2.49, 5.17). Total doses of follicle stimulating hormone (FSH) and luteinizing hormone (LH) during controlled ovarian stimulation (COS) were 2100 IU (IQR: 1800.00, 2700.00) and 1125 IU (IQR: 675.00, 1443.75), respectively, and trigger day E2 levels were 3750.65 pg/mL (IQR: 2622.20, 5204.62) (Table 1).

A median of 17.00 oocytes were recovered from each patient (IQR: 11.00, 24.25), of which 77.47 ± 14.30% were mature (metaphase II; MII). The fertilization, blastulation, and euploidy rates were 81.44 ± 16.29%, 55.62 ± 21.47%, and 60.17 ± 23.72%, respectively. Among the 91.7% (55/60) of participants who underwent an embryo transfer, the implantation rate was 80%, the clinical pregnancy rate was 69.1%, and the live birth rate was 63.6%. Alternatively, 58.3% of the sixty participants achieved the goal of having a live newborn (Table 1).

Slight and inconsistent differences in demographic variables were observed in the comparison between tertiles of essential trace elements across the different biological matrices. In particular, it has been observed that higher FF molybdenum concentrations were associated with an increased likelihood of not having an education beyond high school, while higher zinc levels were more likely to be thinner (Table S1). Additionally, in these exploratory analyses, we are beginning to identify relationships between the evaluated essential trace elements and IVF results. Briefly, we have seen a higher number of total oocytes recovered as the concentration of copper in FF and plasma and the ratio of Cu/Zn and selenium in FF increased. Also, in the upper tertiles of copper and Cu/Zn ratio in FF, a higher concentration of trigger day estradiol (E2) is observed, whereas it is lower with increasing plasma manganese concentration. Also, in plasma, superior zinc concentrations are associated with a higher number of fertilized oocytes and with a lower euploid embryo ratio. Conversely, a higher Cu/Zn ratio is related to a high euploid embryo ratio in this fluid. Finally, a higher concentration of molybdenum in urine obtained on vaginal oocyte retrieval (VOR) day is associated with lower implantation and live newborn ratio (Table S1).

### 2.2. Essential Trace Elements Distribution among Biofluids

Table 2 contains the percentage of samples above the limit of detection and the distributions of essential trace elements in FF, plasma, and urine. Essential trace elements that were undetectable in >50% of the samples were excluded from subsequent analyses (Table 2).

With respect to the FF, moderate–strong positive correlations were observed for the concentrations of copper (r = 0.66), Cu/Zn ratio (r = 0.73), selenium (r = 0.44), and chromium (r = 0.41) in plasma and lithium in urine (r = 0.4) (Figure S1). All other relationships between the trace elements and biofluids were insignificant/negligible (Figure S1). Regarding the relationships between the essential trace elements within each biofluid, we found moderately positive correlations between copper and Cu/Zn ratio (r = 0.57), zinc (r = 0.49), and selenium (r = 0.41) and between lithium and molybdenum (r = 0.46) in the FF and for copper with Cu/Zn ratio (r = 0.8), Cu/Mo (r = 0.53), and of both of these ratios with each other (r = 0.47) in plasma (Figure S2). Conversely, we observed moderate–strong negative associations between zinc and the Cu/Zn ratio (r = −0.34 in FF; r = −0.57 in plasma) and between molybdenum and the Cu/Mo ratio (r = −0.91 in FF; r = −0.78 in plasma) (Figure S2). Further, there were generally moderate–strong correlations among urinary essential trace elements (Figure S2).

### 2.3. Association of Essential Trace Elements Concentrations with Ovarian Response and Preimplantation Outcomes

Following multivariate adjustment for age, BMI, race/ethnicity, and smoking status, the mean differences and relative proportions (95% CI) in reproductive outcomes were evaluated with respect to the essential trace element concentrations modeled as continuous (log-transformed) and presented as the increase among participants in the 20th and 80th percentiles.

The FF copper concentration had statistically significant positive associations with AMH (p20 vs. p80 (95% CI): 4.22 (1.34, 13.25), p trend = 0.015), trigger day E2 (p20 vs. p80 (95% CI): 1.59 (1.28, 1.97), p trend < 0.001), the number of retrieved oocytes (p20 vs. p80 (95% CI): 1.64 (1.25, 2.16), p trend < 0.001), the relative proportion of MII oocytes (p20 vs. p80 (95% CI): 1. 55 (1.16, 2.07), p trend = 0.004), and fertilized embryos (p20 vs. p80 (95% CI): 1.52 (1.11, 2.07), p trend = 0.009). Regarding plasma copper concentration, there were statistically significant positive association with AMH concentration (p20 vs. p80 (95% CI): 4.04 (1.31, 12.50), p trend = 0.016), the number of retrieved oocytes (p20 vs. p80 (95% CI): 1.42 (1.08, 1.86), p trend = 0.012), relative proportion of MII oocytes (p20 vs. p80 (95% CI): 1.34 (1.03, 1.75), p trend = 0.03), and euploid embryos (p20 vs. p80 (95% CI): 1.50 (1.12, 2.01), p trend = 0.007) (Table 3).

Additionally, there were statistically significant positive associations between the FF Cu/Zn ratio and AMH (p20 vs. p80 (95% CI): 3.15 (1.36, 7.31), p trend = 0.009), trigger day E2 (p20 vs. p80 (95% CI): 1.26 (1.05, 1.50), p trend = 0.012), number of retrieved oocytes (p20 vs. p80 (95% CI): 1.24 (1.02, 1.50), p trend = 0.029), and euploid embryos (p20 vs. p80 (95% CI): 1.38 (1.09, 1.75), p trend = 0.009). Similarly, there were statistically significant positive associations between the number of recovered oocytes (p20 vs. p80 (95% CI): 1.37 (1.04, 1.80), p trend = 0.027) and the relative proportion of euploid embryos (p20 vs. p80 (95% CI): 1.38 (1.03, 1.85), p trend = 0.031) with the plasma Cu/Zn ratios. In contrast, the fully adjusted models showed statistically significant negative associations between the Cu/Zn ratio in urine and MII oocytes (p20 vs. p80 (95% CI): 1.38 (1.03, 1.85), p trend = 0.031), fertilized embryos (p20 vs. p80 (95% CI): 1.38 (1.03, 1.85), p trend = 0.031), and blastocysts (p20 vs. p80 (95% CI): 1.38 (1.03, 1.85), p trend = 0.031) (Table 3).

Data was adjusted for age (continuous), BMI (continuous), race/ethnicity, and smoking status (never, ever). The percentile 20 (p20) and 80 (p80) of the essential trace element distributions are presented in Table S2.

A statistically significant positive association was found between plasma manganese concentration and AMH (p20 vs. p80 (95% CI): 5.37 (1.86, 15.53), p trend = 0.003), relative proportion of MII oocytes (p20 vs. p80 (95% CI): 1.31 (1.04, 1.65), p trend = 0.023), fertilized (p20 vs. p80 (95% CI): 1.33 (1.04, 1.70), p trend = 0.022), and euploid embryos (p20 vs. p80 (95% CI): 1.40 (1.10, 1.79), p trend = 0.007). The Cu/Mo ratio in FF also had statistically significant positive associations with euploid embryos (p20 vs. p80 (95% CI): 1.48 (1.01, 2.16), p trend = 0.045) (Table 3).

Also, a statistically significant inverse association was observed between the lithium in FF and number of oocytes retrieved (p20 vs. p80 (95% CI): 0.82 (0.68, 0.98); p trend = 0.03), relative proportion of MII oocytes (p20 vs. p80 (95% CI): 0.80 (0.67, 0.95), p trend = 0.015), and fertilized embryos (p20 vs. p80 (95% CI): 0.78 (0.65, 0.94), p trend = 0.011). (Table 3).

Statistically significant negative associations were observed between the Cu/Mo ratio in urine and the number of oocytes retrieved (p20 vs. p80 (95% CI): 0.83 (0.70, 0.99), p trend = 0.041), the relative proportion of MII oocytes (p20 vs. p80 (95% CI): 0.83 (0.70, 0.98), p trend = 0.027), fertilized embryos (p20 vs. p80 (95% CI): 0.84 (0.70, 1.00), p trend = 0.047), and blastocysts (p20 vs. p80 (95% CI): 0.83 (0.69, 0.99), p trend = 0.035) in addition to the plasma selenium and urinary iron with E2 (p20 vs. p80 (95% CI): 0. 86 (0.74, 0.99), p trend = 0.038; and 0.82 (0.68, 0.98), p trend = 0.028; respectively) (Table 3).

### 2.4. Association of Essential Trace Elements Concentrations with Clinical IVF Outcomes

**Figure 1:**
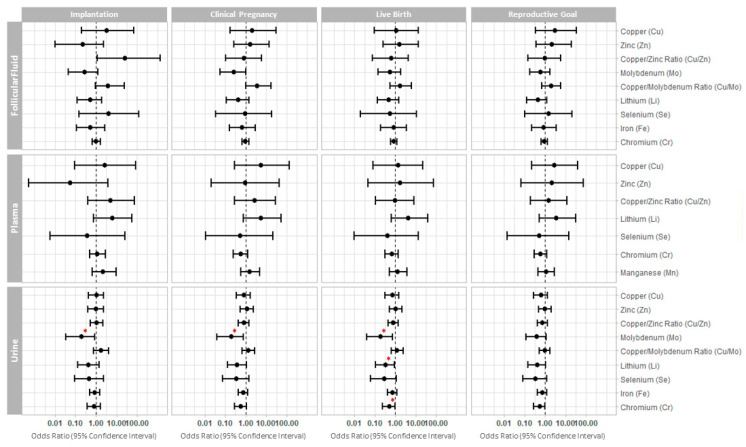
Forest plot representing the odds ratios (95% confidence interval) for clinical reproductive outcomes. The odds ratios (95% confidence interval) are presented for implantation, clinical pregnancy, live birth, and reproductive goal (achievement of a live birth in a given cycle) following single frozen euploid embryo transfer, across the essential trace elements quantified in each biofluid. * *p* < 0.05.

We similarly examined the relationships between the essential trace element concentrations in FF, plasma, and urine with clinical IVF outcomes using multivariate models adjusted for age, BMI, race/ethnicity, and smoking status (Figure 1 and Table S3).

Higher urinary molybdenum concentration was significantly associated with a lower probability of implantation (OR (95% CI): 0.19 (0.03, 0.86), p value = 0.043), clinical pregnancy (OR (95% CI): 0.2 (0.04, 0.76), *p* value = 0.027), and live birth (OR (95% CI): 0.2 (0.04, 0.72), *p* value = 0.024). Further, lower probability of a live birth was also significantly associated with elevated concentrations of lithium (OR 0.33; 95% CI: 0.11, 0.89; *p* value = 0.036) and chromium (OR 0.5; 95% CI: 0.24, 0.97; *p* value = 0.049) in urine (Figure 1 and Table S3). We did not observe significant differences in the probabilities of IVF clinical outcomes associated with the concentrations of essential trace elements measured in the FF or plasma (Figure 1 and Table S3).

## 3. Discussion

This study investigated the relationships between several essential trace elements in distinct biofluids and IVF outcomes following ICSI, PGT-A, and SET/FET, to identify excessive exposures and assess how these environmental factors affect reproduction. Our prospective design reduced the likelihood of reverse causality, with delayed embryo transfers to avoid the effects of COS on endometrial function, and PGT-A to ensure the transfer of a euploid embryo.

Overall, the essential trace element concentrations that we observed in the FF and urine corroborate with previous findings [11], but the data presented herein evidenced that FF, and particularly, plasma, are more suitable for trace element screening and better for predicting intermediate reproductive outcomes than urine (where excess excretion needs to be considered). Similar to previous studies [14,15,16], we have observed that urine (reflecting essential trace element clearance) has a distinct metabolic signature than FF and plasma (which reflect essential trace element homeostasis), which could help explain the different associations observed for each biological fluid.

Other groups have evaluated how trace elements in different biofluids from women undergoing IVF treatments impact their reproductive outcomes [11,12,13,17,18,19], however, these studies were performed in general IVF populations with potentially confounding factors (e.g., aneuploid transfers and the effect of endometrial stimulation hormones), leading to inconsistent findings. Notably, essential trace elements were scarcely investigated in plasma, and, to our knowledge, this is the first report associating lithium (in any biofluid) to reproductive outcomes. Finally, we highlight that none of these studies were as extensive as ours, which simultaneously examined three biofluids and eight essential trace elements for each patient.

Copper is an essential trace element that acts as a structural and/or regulatory cofactor of numerous proteins, including several redox-related enzymes (e.g., superoxide dismutase, amino acid oxidase, cytochrome c oxidase, and dopamine β-hydroxylase ceruloplasmin) and neuropeptides (including some involved in the hypothalamic–pituitary–gonadal axis) [4,20,21]. Dietary sources of copper include liver, mussels, oysters, whole grains, nuts, and chocolate, and copper deficiency was associated with disrupted cholesterol metabolism [4] and compromised fertility in animals [22]. The influence of copper on the reproductive outcomes of women undergoing IVF has been evaluated in previous studies [11,12,17,18]. Ingle et al. [11] observed a significant increase in the total number of oocytes recovered in relation to urinary copper concentrations while Sun et al. [18] observed a significantly positive correlation between FF copper concentration and oocyte maturation and fertilization rates. These results are consistent with our observations that elevated copper concentrations in FF and plasma improved ovarian response and preimplantation outcomes. Taken together, these findings suggest that an optimal concentration of copper in the ovarian microenvironment may promote oocyte maturation and developmental competence, possibly by mediating the secretion of reproduction-related hormones [20,21] or directly improving oocyte quality, as was previously demonstrated with copper supplementation during in vitro maturation [23,24].

Zinc is an essential trace element required as a cofactor in more than 300 enzymes, with a key role in both the synthesis and maintenance of genetic material and the metabolism of proteins, lipids, and carbohydrates, therefore, playing an integral part in cell division. Cereals and meat are the main zinc sources [4]. Our patients had zinc concentrations similar to those described in other studies using IVF patients [11,13]. Although the FF concentrations were lower than previously reported [17,18], this discrepancy is likely due to copper and zinc concentrations being lower in women undergoing IVF treatment than in the general population [8,9,10]. Moreover, the Cu/Zn ratio is considered to have more clinical relevance than the concentration of either copper or zinc alone, due to the possibility of disrupting their antagonistic interactions with slight variation in their concentrations [25,26]. Indeed, our results showed that a higher Cu/Zn ratio in FF and plasma was associated with higher rates of oocyte retrieval and euploid embryos, but the elevated urinary Cu/Zn ratios, reflecting excess urinary elimination, were associated with poor oocyte retrieval and preimplantation outcomes. This finding not only highlights the importance selecting the right biofluid to detect exposure to these elements but also understanding the underlying physiological processes to correctly interpret these contradicting results.

Molybdenum participates as a cofactor in several metalloenzymes with oxidoreductase activity [4,27]. These enzymes are involved with heterocyclic compounds (e.g., purines and pyrimidines), sulfur amino acids catabolism, aromatic aldehyde, along with drug and toxin metabolism [4,27]. Molybdenum is mainly consumed through dietary sources, including legumes, cereal grains, leafy greens, milk, organ meat (e.g., liver, kidney), and nuts [4].

Molybdenum’s effect on reproduction has been mainly studied in relation to male fertility [27,28,29], but its impact on female reproduction remains elusive. Ingle et al. [11] evaluated molybdenum in the FF and urine of women undergoing IVF and found a significant increase in the total number of oocytes recovered associated with higher concentrations of molybdenum in urine but not in FF; no other associations were found with reproductive outcomes. We also observed positive trends between urinary molybdenum concentration and the number of oocytes recovered in our study; however, they did not reach statistical significance, potentially due to variations in stimulation protocols, interaction with other essential trace elements, or the need for a larger sample size to discern the effect of this association. Additional evidence from a murine model suggests that oral molybdenum exposure impairs estrous activity and worsens embryogenesis, possibly by dysregulating copper cofactor enzymes [30]. Taken together with our findings of elevated urinary molybdenum on the day of VOR significantly decreasing the probability of implantation, clinical pregnancy, live birth, and similar trends in FF, this evidence reinforces that molybdenum is potentially detrimental to female fertility.

Like zinc, molybdenum antagonizes copper. Thus, excessive molybdenum consumption could induce copper deficiency [27,31] while elevated copper levels decreased molybdenum toxicity [28,29]. Based on this interplay, we incorporated the Cu/Mo ratio in our analyses. Our results showed a statistically significant negative association of the urinary Cu/Mo ratio with the response to ovarian stimulation and IVF embryological variables. These results reinforce the theory that the relationship between closely related elements such as copper, molybdenum, and zinc may be more informative than its absolute concentrations. Notably, this approach has not been used in other studies evaluating these elements and may help to explain the discrepancies between studies.

Manganese is a cofactor of numerous enzymes, including some that modulate steroidogenesis, and acts as a component of antioxidant enzymes (e.g., manganese superoxide dismutase) [4,32]. Dietary manganese is present in green vegetables, nuts, cereals, and tea [4]. A deficient daily intake (<1.8 mg) was associated with an increased risk of anovulation [33], which was consistent with our findings that higher plasma manganese concentrations were related to better follicular response and preimplantation outcomes. Ingle et al. [11] also recovered more MII oocytes in women with higher urinary manganese concentrations; however, higher FF manganese concentrations were associated with lower oocyte maturation rates. Unfortunately, in our population, the manganese concentration was below the detection limit in most urine and FF samples (Table 2); thus, it was not possible to evaluate these associations reliably.

Lithium is naturally present in all human organs and tissues and can be obtained from dietary sources (e.g., grains, vegetables, or water) and is utilized in psychopharmacology [34,35,36]. Lithium is absorbed in the intestinal tract, distributed in body fluids, and excreted through the kidneys [34]. Whether lithium is an essential trace element remains controversial [34]. We included it to address the research gap regarding its impact on female reproduction as some reports suggested that increased lithium exposure has undesirable side effects on female reproduction [37,38,39,40,41]. Indeed, lithium chloride inhibited folliculogenesis, promoted follicular atresia, and impaired embryo development in animal models [38,39,41], and both in vitro and in vivo models demonstrated these effects that are mediated by its ability to interfere with steroidogenesis [37,39,40]. Accordingly, our data suggests elevated lithium levels impair human folliculogenesis and oocyte competence, at non-pharmacological concentrations. In addition, higher environmental lithium exposure was associated with embryonic developmental defects, and women taking lithium-based medication during pregnancy had an increased likelihood of miscarriage [42,43]. These findings support our observations of a lower probability of live birth in participants with elevated urinary lithium concentrations. In view of this evidence, we consider both pharmacological and natural lithium management important [44] and to be able to identify potentially hidden exposures and establish the minimum effective concentration that does not hamper reproductive functions.

Chromium is a trace element that can be found in different oxidation states, with op-posing effects on human physiology. For example, trivalent chromium plays an essential role in glucose, carbohydrate, and lipid metabolism. In contrast, hexavalent chromium has no physiological role, but its exposure is considered carcinogenic due to risks of oxidative or DNA damage, chromosomal instability, and cell death [4]. Studies evaluating chromium in different biological fluids from women undergoing IVF have generally found no association with embryological variables or IVF clinical outcomes [11,15,16]. Regarding ovarian stimulation response, FF cadmium is reported inconsistently [11,16], and no associations have been found for serum cadmium [15,16]. In our study, we observed that elevated urinary chromium concentration is associated with a lower probability of live birth. This may be explained by chromium affecting embryonic development as excessive exposure was associated with fetal growth retardation and preterm birth [45,46].

Selenium is a glutathione peroxidase cofactor and plays a central role in the body’s antioxidant defenses [4]. Discrepancies have been reported among studies evaluating selenium concentrations in relation to reproductive outcomes. Some authors have reported that higher serum and hair selenium concentrations were associated with higher numbers of total and mature oocytes recovered [47,48], while others did not observe such associations with serum and FF selenium [15]. Despite elevated FF selenium being associated with accelerated embryo development but lower blastulation rates, there are no reported associations between serum selenium and embryo development [12,15,47]. Furthermore, Wdowiak et al. found a positive association between FF selenium and the clinical pregnancy rate [12], no other associations have been observed between FF or serum selenium and IVF clinical outcomes [15,47]. In our cohort, serum selenium only had a negative association with trigger day E2.

In addition to its role as an oxygen transporter, iron is a constituent of several enzymes (e.g., catalase, peroxidase, and cytochromes) involved in energy production [4]. Our findings were consistent with those of previous studies reporting no associations be-tween FF iron and IVF treatment outcomes, including ovarian reserve parameters, embryology or clinical IVF outcomes [12,17], and consistent with our findings.

Finally, the limitations of our study include that this was a pilot study with a relatively small sample size and, thus, a limited statistical power. Further, although we did not measure the paternal levels of trace elements, we acknowledge their potential to impact embryonic development through the sperm’s epigenome, as Finke et al. [49] recently linked trace element deficiency to genomic instability and low-grade inflammation in male mice. Although Finke’s study did not specifically analyze reproductive tissues, these elements can alter gene expression through epigenetic regulation [50].

Thus, studies with larger cohorts, including other environmental factors such as non-essential trace elements, are needed to confirm these preliminary findings and to evaluate potential interactions between analytes.

## 4. Materials and Methods

### 4.1. Study Population

Sixty women (aged 18–42) undergoing ICSI with PGT-A and SET/FET, between July 2018 and November 2019, at RMANJ—Basking Ridge (USA) were enrolled in this prospective, single-center, pilot study. Patients were excluded from the study if they presented with severe male factor infertility or had untreated systemic or endocrine disorders, abnormal karyotypes, thrombophilia, an atypical uterus, irregular endometrium, or endometrium measuring <7 mm on the day of embryo transfer. Women who took part in the trial received the same clinical and laboratory care as if they had not taken part in the study.

### 4.2. Collection of Follicular Fluid, Plasma, and Urine Samples

Urine was collected from fasting participants on the morning of vaginal oocyte retrieval (VOR) in sterile polypropylene containers and stored at 4 °C. Urine samples were centrifuged at 500× *g* for 7 min to pellet the sediment and collect the supernatant (urine), which was aliquoted and stored at −80 °C. Blood samples were also collected on the VOR day, from fasting participants, in EDTA tubes. These samples were centrifuged at 1300× *g* for 15 min at 4 °C, to isolate the plasma which was aliquoted and stored at −80 °C. Following isolation of the cumulus–oocyte complexes immediately after VOR, the FF from the aspirates of each patient was pooled and centrifuged at 1000× *g* for 3 min to remove cellular debris, aliquoted, and stored at −80 °C. Once all the study samples were obtained, they were sent to IVI Foundation (Valencia, Spain) on dry ice and transferred to the Mass Spectrometry and Geochemical Applications unit of the CIEMAT (Madrid, Spain) for essential trace element quantification. Upon arrival at the CIEMAT, the samples were stored at −80 °C until further analysis.

### 4.3. Quantification of Essential Trace Elements Using ICP-MS

The concentrations of the essential trace elements in human FF, plasma, and urine were measured with inductively coupled plasma mass spectrometry (ICP-MS), using an i-CapRQ mass spectrometer (Thermo Fisher Scientific, Madrid, Spain) with a quadrupole analyzer and dual mode secondary electron multiplier (SEM) as a detection system. Except for the lithium quantification, the analysis of the remaining trace elements incorporated a collision cell (CCT) with kinetic energies discrimination (KED) mode to attenuate the polyatomic interference of the biofluids.

The day before ICP-MS, samples were defrosted and refrigerated at 4 °C. To prepare samples for ICP-MS, 0.5 mL of each FF sample was diluted 1:20, in 0.5% (*v*/*v*) HNO_3_ (distilled in situ using a DuoPUR Sub-boiling Distillation System (Milestone Inc., Madrid, Spain) and 0.0005% (*v*/*v*) Triton^®^ X-100 (Sigma-Aldrich, Madrid, Spain), as surfactant, including 1 μg/L gallium (Ga), indium (In), and lutetium (Lu) as internal standards (SPEX CertiPrep, Thermo Fisher Scientific, Madrid, Spain; and Inorganic Ventures, Isostandards, Madrid, Spain). Alternatively, 0.5 mL of each plasma sample was digested in a DigiPrep block (SCP SCIENCE, Quebec, QC, Canada) with temperature ramping, using 2 mL of 65% HNO_3_ and 0.1 mL of 40% *w*/*v* Suprapur^®^ grade hydrofluoric acid (at 75 °C for 15 min) (Merck Millipore, Madrid, Spain), followed by 1 mL of H_2_O_2_ 30% *v*/*v* (at 115 °C for 60 min) (Merck Millipore, Madrid, Spain). Digested plasma samples were topped to 10 mL with Milli-Q water (18.2 MΩ/cm) (Merck Millipore, Madrid, Spain) after adding 1 μg/L Ga, In, and Lu (SPEX CertiPrep, Thermo Fisher Scientific, Madrid, Spain; and Inorganic Ventures, Isostandards, Madrid, Spain). On the other hand, 0.5 mL of each urine sample was diluted 1:10 in 2% (*v*/*v*) HNO_3_ with 1 μg/L Ga, In, and Lu (SPEX CertiPrep, Thermo Fisher Scientific, Madrid, Spain; and Inorganic Ventures, Isostandards, Madrid, Spain).

An external calibration method, including calibration standards from 0.01 to 500 µG/L daily prepared by serial dilution, was used during quantification. Blanks and calibration standards (ranging from 0.01–500 µg/L) (SPEX CertiPrep, Thermo Fisher Scientific, Madrid, Spain; and Inorganic Ventures, Isostandards, Madrid, Spain) prepared in 2% (*v*/*v*) HNO_3_ for plasma and urine samples, or 0.5% (*v*/*v*) HNO_3_ and 0.0005% (*v*/*v*) Triton^®^ X-100 for FF samples. Note, they both had a final concentration of 1 µg/L Ga, In, and Lu as internal standards.

To correct for dilution of the essential trace element concentrations in urine, we normalized our findings to creatinine measured in the aliquoted urine (preserved at −80 °C), using a commercial kit (Creatinine Parameter Assay Kit, Cat. # KGE005, Bio-Techne R&D Systems, Madrid, Spain).

### 4.4. Clinical Management and Outcome Assessment

Patient baseline characteristics (i.e., date of birth, weight, and height) were collected at enrollment to calculate age and body mass index (BMI; kg/m^2^). Other demographic variables, such as race/ethnicity, education, or smoking were self-reported on a questionnaire. The patients’ most recent serum anti-Müllerian hormone (AMH) levels and reproductive outcomes were retrieved from their electronic medical records, while trigger day serum estradiol (E2) was measured in blood, using an in-clinic automated electrochemiluminescence immunoassay.

All patients underwent controlled ovarian stimulation (COS) employing a gonadotropin releasing hormone (GnRH) antagonist protocol in the luteal phase. The clinician’s judgment and assessment of the participant’s ovarian reserve were used to determine the gonadotrophin doses. When follicles reached a diameter of 15–20 mm, human chorionic gonadotropin (hCG) and/or a GnRH-agonist trigger was administered for final oocyte maturation, followed by ultrasound-guided VOR 36 h later [51].

Cumulus cells were removed from the oocytes, and the number and maturation of recovered oocytes was recorded. ICSI was performed to reduce the risk of DNA contamination during PGT-A and standardize fertilization processes. The number of fertilized oocytes was determined 18 h after ICSI, and sequential culture medium was utilized to culture embryos to the blastocyst stage [51]. Trophectoderm biopsies for PGT-A were collected prior to embryo vitrification [51].

A single euploid embryo was transferred following endometrial priming using oral estrogen and intramuscular progesterone [51]. Embryos were thawed and placed into the endometrial cavity using a catheter under ultrasound guidance on the day of substitution.

Internal protocols were followed for evaluating the clinical IVF outcomes. Serum hCG levels > 6 mIU/mL approximately 14 days after embryo transfer were used to define implantation. Clinical pregnancies were confirmed using ultrasonography, and live births were defined by a neonate born after 24 weeks of gestation.

### 4.5. Statistical Analyses

All statistical analyses were performed using R software (version 3.6.2). Participants’ baseline demographic and reproductive characteristics were presented as median ± interquartile ranges (IQR) or percentages. Using Kruskal–Wallis tests for continuous variables and chi-square testing for categorical variables, associations between essential trace element concentrations and baseline demographic and reproductive variables were assessed with the “tableone” package [52]. The relationships of the distinct essential trace elements within and between each biofluid were assessed using correlation matrices generated with the “corrplot” package [53], while their relationships with the IVF outcomes were analyzed using generalized linear multivariate mixed models with random intercepts. We estimated the mean differences for AMH and E2 concentrations using Gaussian distribution, while those for discrete count variables (such as total number of retrieved oocytes, relative proportion of mature (metaphase II; MII) oocytes (offset by the total number of oocytes retrieved), relative proportion of fertilized embryos (offset by the number of MII oocytes), relative proportion of blastocyst (offset by the number of fertilized embryos), and relative proportion of euploid embryos (offset by the number of blastocyst)), were calculated using Poisson distributions. Essential trace elements concentrations were modeled as continuous (log-transformed), and linear associations were obtained by comparing the increase between the 20th and 80th percentile. Finally, a binomial distribution generated with the “questionr” package [54] was used to calculate the odds ratios (OR) for clinical IVF outcomes (i.e., implantation, clinical pregnancy, and live birth) relative to embryo transfers and reproductive goals (probability of live birth for each treatment started).

For better interpretation of the results, the marginal population means adjusted for all the covariates of the model are presented. Variables measured as potential confounders included factors previously related to reproductive outcomes and essential trace element exposition [55]. Final models were adjusted for age (continuous), BMI (continuous), race/ethnicity, and smoking status (never, ever). A statistical significance level of 0.05 was set for all tests.

## 5. Conclusions

This study highlighted that essential trace element concentrations influence clinical outcomes of women undergoing ICSI with PGT-A and SET/FET. The plasma manganese concentration along with the copper concentration and copper/zinc ratio, both in the FF and plasma, were positively correlated with ovarian response and preimplantation outcomes. Alternatively, elevated FF lithium concentrations were significantly associated with compromised oocyte maturation and competence, while elevated urinary molybdenum concentrations were related to poor implantation, clinical pregnancy, and live birth. Larger study cohorts are needed to validate these preliminary findings and additional preclinical models are needed to establish safe ranges of essential trace element concentrations for ensuring optimal reproductive function. Finally, our associations could provide a foundation for future studies aiming to assess whether copper and manganese supplementation (either through dietary recommendations or dietary supplements) could effectively improve reproductive outcomes in both animal and human models.

## Figures and Tables

**Table 1:** Baseline characteristics of the participating women (n = 60).

**Demographic Characteristics**	
Age (y), median (IQR)	33.40 [31.37, 36.50]
Body mass index (kg/m^2^), median (IQR)	23.87 [21.57, 26.30]
Race/ethnic group, n (%)	
White/Caucasian	43 (71.70%)
Afro-American	2 (3.30%)
Asian	6 (10.00%)
Hispanic	6 (10.00%)
Other	3 (5.00%)
Education, n (%)	
>High school	55 (94.80%)
Smoking, n (%)	
Never Smoker	49 (81.70%)
Ex-smoker	10 (16.70%)
Passive smoker	1 (1.70%)
**Reproductive Characteristics**	
Anti-Müllerian hormone (ng/mL), median (IQR)	3.60 [2.49, 5.17]
Initial treatment protocol, n (%)	
GnRH Antagonist	60 (100.0%)
Total FSH dose during stimulation (IU), median (IQR)	2100.00 [1800.00, 2700.00]
Total LH dose during stimulation (IU), median (IQR)	1125.00 [675.00, 1443.75]
Estradiol trigger levels (pg/mL), median (IQR)	3750.65 [2622.20, 5204.62]
Number of retrieved oocytes, median (IQR)	17.00 [11.00, 24.25]
Mature (MII) oocyte rate, % mean ± SD	77.47 ± 14.30%
Fertilization rate, % mean ± SD	81.44 ± 16.29%
Blastulation rate, % mean ± SD	55.62 ± 21.47%
Euploid rate, % mean ± SD	60.17 ± 23.72%
Transfer rate, n (%)	55 (91.70%)
Implantation (positive hCG) rate, n (%)	44 (80.00%)
Clinical pregnancy rate, n (%)	38 (69.10%)
Live birth rate, n (%)	35 (63.60%)
Goal rate, n (%)	35 (58.30%)

Note: the goal rate refers to the achievement of a live birth within a given cycle.

**Table 2:** Distribution of essential trace elements concentrations in follicular fluid, plasma, and urine.

	LOD	Samples below the LOD (%)	GM (SD)	Minimum	25%	50%	75%	Maximum
**Follicular Fluid**								
Copper (Cu) (ng/mL)	NA	0%	974.36 (277.77)	535.00	825.00	966.50	1210.00	1970.00
Zinc (Zn) (ng/mL)	NA	0%	383.49 (109.61)	81.71	340.98	392.43	454.23	690.00
Copper/Zinc Ratio (Cu/Zn)			2.54 (1.39)	1.17	2.14	2.54	2.94	11.60
Molybdenum (Mo) (ng/mL)	1	32%	1.06 (0.60)	0.50	0.50	1.22	1.71	2.50
Copper/Molybdenum Ratio (Cu/Mo)			935.95 (745.88)	377.50	564.70	898.84	1525.00	3940.00
Lithium (Li) (ng/mL)	1	18%	1.34 (1.98)	0.50	1.10	1.35	1.78	13.00
Selenium (Se) (ng/mL)	NA	0%	66.30 (14.09)	31.00	60.50	69.00	73.50	111.00
Iron (Fe) (ng/mL)	NA	0%	539.37 (535.84)	278.00	429.46	487.60	598.32	3455.00
Chromium (Cr) (ng/mL)	1	50%	2.65 (11.57)	0.50	0.50	0.84	12.50	62.36
Manganese (Mn) (ng/mL)	1	59.60%	0.76 (0.51)	0.50	0.50	0.50	1.20	2.20
**Plasma**								
Copper (Cu) (ng/mL)	NA	0%	1241.62 (274.66)	801.41	1107.79	1279.68	1434.14	2030.92
Zinc (Zn) (ng/mL)	NA	0%	960.58 (150.10)	714.03	847.36	962.61	1062.44	1281.21
Copper/Zinc Ratio (Cu/Zn)			1.29 (0.35)	0.67	1.09	1.33	1.61	2.13
Molybdenum (Mo) (ng/mL)	5	71.90%	3.29 (2.20)	2.50	2.50	2.50	5.26	11.64
Copper/Molybdenum Ratio (Cu/Mo)			379.31 (170.55)	112.45	277.92	452.52	550.59	812.37
Lithium (Li) (ng/mL)	5	8.50%	5.91 (1.42)	2.50	5.72	6.27	6.71	10.08
Selenium (Se) (ng/mL)		0%	99.43 (18.20)	60.90	91.53	99.08	106.56	185.52
Chromium (Cr) (ng/mL)	5	47.50%	5.24 (8.14)	2.50	2.50	5.36	8.92	52.36
Manganese (Mn) (ng/mL)	5	45.80%	4.30 (5.10)	2.50	2.50	4.12	7.05	38.86
**Urine**								
Copper (Cu) (ng/mL)	5	5.20%	5.38 (5.65)	0.25	2.68	6.60	11.35	25.55
Creatinine Corrected (ug/g CR)			6.18 (6.52)	0.14	1.96	4.68	8.23	10.49
Zinc (Zn) (ng/mL)	50	5.20%	270.87 (354.78)	25.00	158.63	303.77	494.93	1726.10
Creatinine Corrected (ug/g CR)			310.95 (635.96)	26.84	138.93	204.58	323.81	473.12
Copper/Zinc Ratio (Cu/Zn)			0.02 (0.05)	0.00	0.01	0.02	0.04	0.31
Molybdenum (Mo) (ng/mL)	5	1.70%	46.25 (51.62)	2.50	34.50	53.81	81.08	311.68
Creatinine Corrected (ug/g CR)			53.09 (43.55)	12.04	24.66	38.24	54.24	74.78
Copper/Molybdenum Ratio (Cu/Mo)			0.12 (0.18)	0.01	0.08	0.13	0.18	1.02
Lithium (Li) (ng/mL)	5	1.70%	23.32 (28.16)	2.50	13.72	23.26	36.81	173.50
Creatinine Corrected (ug/g CR)			26.77 (26.89)	8.37	12.82	17.13	25.66	35.21
Selenium (Se) (ng/mL)	NA	0%	39.74 (30.55)	4.32	32.35	44.49	63.95	157.56
Creatinine Corrected (ug/g CR)			45.62 (27.53)	17.300	27.040	35.890	40.920	58.080
Iron (Fe) (ng/mL)	5	6.90%	19.22 (46.12)	2.50	19.00	27.00	27.50	365.24
Creatinine Corrected (ug/g CR)			22.07 (243.62)	1.440	5.150	12.690	20.830	37.080
Chromium (Cr) (ng/mL)	0.5	56.90%	0.41 (0.58)	0.25	0.25	0.25	0.65	3.54
Creatinine Corrected (ug/g CR)			0.47 (0.80)	0.070	0.130	0.230	0.460	0.800
Manganese (Mn) (ng/mL)	0.5	91.40%	0.28 (0.16)	0.25	0.25	0.25	0.25	1.11
Creatinine Corrected (ug/g CR)			0.32 (0.65)	0.070	0.130	0.180	0.250	0.470

Abbreviations: CR, Creatinine; GM, Geometric Mean; LOD, Limit of Detection; SD, Standard Deviation.

**Table 3:** Mean differences (95% CI) for ovarian response-related and preimplantation IVF outcomes by essential trace element concentrations.

	Anti-Müllerian Hormone		Trigger Day Estradiol		Number of Retrieved Oocytes		Relative Proportion of Mature (MII) Oocytes		Relative Proportion of Fertilized Embryos		Relative Proportion of Blastocysts		Relative Proportion of Euploid Embryos	
	p20 vs. p80 (95% CI)	p Trend	p20 vs. p80 (95% CI)	p Trend	p20 vs. p80 (95% CI)	p Trend	p20 vs. p80 (95% CI)	p Trend	p20 vs. p80 (95% CI)	p Trend	p20 vs. p80 (95% CI)	p Trend	p20 vs. p80 (95% CI)	p Trend
**Follicular Fluid**														
Copper (Cu)	**4.22 (1.34, 13.25)**	**0.015**	**1.59 (1.28, 1.97)**	**<0.001**	**1.64 (1.25, 2.16)**	**<0.001**	**1.55 (1.16, 2.07)**	**0.004**	**1.52 (1.11, 2.07)**	**0.009**	1.35 (0.97, 1.88)	0.074	1.26 (0.92, 1.73)	0.152
Zinc (Zn)	0.79 (0.34, 1.81)	0.566	1.05 (0.88, 1.25)	0.569	1.06 (0.85, 1.33)	0.582	1.04 (0.84, 1.28)	0.719	1.03 (0.83, 1.28)	0.759	1.08 (0.87, 1.36)	0.469	0.89 (0.71, 1.12)	0.324
Copper/Zinc Ratio (Cu/Zn)	**3.15 (1.36, 7.31)**	**0.009**	**1.26 (1.05, 1.50)**	**0.012**	**1.24 (1.02, 1.50)**	**0.029**	1.21 (1.00, 1.47)	0.055	1.19 (0.97, 1.46)	0.093	1.08 (0.86, 1.35)	0.513	**1.38 (1.09, 1.75)**	**0.009**
Molybdenum (Mo)	0.78 (0.18, 3.44)	0.741	1.10 (0.78, 1.56)	0.560	1.04 (0.68, 1.58)	0.868	1.02 (0.67, 1.55)	0.938	0.95 (0.61, 1.47)	0.809	1.01 (0.65, 1.58)	0.952	0.84 (0.54, 1.30)	0.414
Copper/Molybdenum Ratio (Cu/Mo)	3.16 (0.87, 11.47)	0.079	1.17 (0.86, 1.59)	0.297	1.29 (0.90, 1.85)	0.16	1.27 (0.89, 1.83)	0.183	1.33 (0.91, 1.94)	0.138	1.24 (0.84, 1.83)	0.264	**1.48 (1.01, 2.16)**	**0.045**
Lithium (Li)	0.56 (0.27, 1.18)	0.124	1.02 (0.87, 1.20)	0.808	**0.82 (0.68, 0.98)**	**0.03**	**0.80 (0.67, 0.95)**	**0.015**	**0.78 (0.65, 0.94)**	**0.011**	0.89 (0.73, 1.08)	0.223	0.89 (0.73, 1.07)	0.216
Selenium (Se)	0.93 (0.39, 2.20)	0.867	1.08 (0.90, 1.29)	0.408	1.08 (0.87, 1.35)	0.488	1.02 (0.82, 1.26)	0.879	0.99 (0.79, 1.23)	0.895	0.96 (0.77, 1.19)	0.698	0.88 (0.74, 1.07)	0.19
Iron (Fe)	0.81 (0.39, 1.67)	0.564	1.01 (0.87, 1.17)	0.923	0.99 (0.83, 1.19)	0.909	0.96 (0.81, 1.14)	0.626	0.96 (0.80, 1.15)	0.63	0.90 (0.75, 1.09)	0.287	0.89 (0.74, 1.07)	0.218
Chromium (Cr)	0.84 (0.20, 3.54)	0.813	1.08 (0.80, 1.44)	0.618	1.16 (0.81, 1.66)	0.42	1.01 (0.70, 1.46)	0.946	0.92 (0.63, 1.35)	0.658	0.97 (0.66, 1.43)	0.882	0.89 (0.62, 1.30)	0.549
**Plasma**														
Copper (Cu)	**4.04 (1.31, 12.50)**	**0.016**	1.22 (0.96, 1.56)	0.108	**1.42 (1.08, 1.86)**	**0.012**	**1.34 (1.03, 1.75)**	**0.03**	1.32 (1.00, 1.75)	0.053	1.34 (0.99, 1.81)	0.056	**1.50 (1.12, 2.01)**	**0.007**
Zinc (Zn)	1.68 (0.55, 5.13)	0.354	0.92 (0.73, 1.16)	0.473	0.95 (0.71, 1.26)	0.711	0.96 (0.72, 1.27)	0.759	1.03 (0.76, 1.39)	0.861	1.12 (0.82, 1.54)	0.465	0.99 (0.71, 1.37)	0.936
Copper/Zinc Ratio (Cu/Zn)	2.20 (0.70, 6.93)	0.174	1.23 (0.97, 1.56)	0.086	**1.37 (1.04, 1.80)**	**0.027**	1.29 (0.99, 1.68)	0.059	1.23 (0.93, 1.63)	0.151	1.18 (0.88, 1.59)	0.271	**1.38 (1.03, 1.85)**	**0.031**
Lithium (Li)	1.51 (1.00, 2.29)	0.052	1.01 (0.92, 1.11)	0.834	0.97 (0.88, 1.08)	0.584	0.97 (0.88, 1.06)	0.469	0.99 (0.89, 1.10)	0.86	1.04 (0.93, 1.17)	0.5	1.10 (0.97, 1.25)	0.131
Selenium (Se)	1.12 (0.55, 2.27)	0.756	**0.86 (0.74, 0.99)**	**0.038**	0.96 (0.81, 1.15)	0.652	0.96 (0.81, 1.15)	0.669	0.97 (0.81, 1.17)	0.769	1.01 (0.84, 1.23)	0.882	1.05 (0.87, 1.26)	0.602
Iron (Fe)	1.82 (0.68, 4.85)	0.224	0.98 (0.79, 1.20)	0.825	1.04 (0.81, 1.34)	0.746	1.13 (0.89, 1.44)	0.299	1.17 (0.91, 1.51)	0.214	1.13 (0.87, 1.48)	0.352	1.06 (0.82, 1.38)	0.639
Chromium (Cr)	2.19 (0.70, 6.88)	0.174	1.13 (0.89, 1.44)	0.314	1.24 (0.93, 1.65)	0.148	1.24 (0.94, 1.63)	0.118	1.26 (0.94, 1.68)	0.12	1.09 (0.79, 1.49)	0.595	1.10 (0.81, 1.51)	0.535
Manganese (Mn)	**5.37 (1.86, 15.53)**	**0.003**	1.16 (0.91, 1.47)	0.227	1.28 (0.98, 1.66)	0.066	**1.31 (1.04, 1.65)**	**0.023**	**1.33 (1.04, 1.70)**	**0.022**	1.27 (0.97, 1.65)	0.082	**1.40 (1.10, 1.79)**	**0.007**
**Urine**														
Copper (Cu)	0.87 (0.42, 1.79)	0.695	0.94 (0.81, 1.09)	0.397	0.90 (0.77, 1.06)	0.213	0.91 (0.78, 1.06)	0.225	0.90 (0.77, 1.06)	0.204	0.91 (0.78, 1.08)	0.272	0.92 (0.78, 1.08)	0.305
Zinc (Zn)	1.16 (0.49, 2.75)	0.729	1.03 (0.86, 1.23)	0.729	1.11 (0.90, 1.38)	0.32	1.12 (0.92, 1.38)	0.255	1.14 (0.92, 1.41)	0.223	1.15 (0.93, 1.43)	0.189	1.06 (0.84, 1.34)	0.595
Copper/Zinc Ratio (Cu/Zn)	0.75 (0.29, 1.92)	0.539	0.91 (0.75, 1.10)	0.312	0.81 (0.65, 1.01)	0.057	**0.81 (0.65, 0.99)**	**0.045**	**0.78 (0.63, 0.98)**	**0.032**	**0.79 (0.63, 0.99)**	**0.039**	0.84 (0.66, 1.08)	0.171
Molybdenum (Mo)	1.97 (0.74, 5.27)	0.172	1.13 (0.92, 1.39)	0.223	1.16 (0.93, 1.44)	0.195	1.20 (0.97, 1.48)	0.093	1.15 (0.92, 1.44)	0.221	1.22 (0.97, 1.52)	0.083	1.11 (0.88, 1.41)	0.371
Copper/Molybdenum Ratio (Cu/Mo)	0.63 (0.28, 1.38)	0.238	0.88 (0.75, 1.04)	0.125	**0.83 (0.70, 0.99)**	**0.041**	**0.83 (0.70, 0.98)**	**0.027**	**0.84 (0.70, 1.00)**	**0.047**	**0.83 (0.69, 0.99)**	**0.035**	0.86 (0.71, 1.04)	0.119
Lithium (Li)	1.37 (0.48, 3.93)	0.553	1.10 (0.89, 1.37)	0.376	0.92 (0.72, 1.19)	0.53	0.96 (0.76, 1.21)	0.717	0.94 (0.73, 1.20)	0.597	1.09 (0.85, 1.38)	0.487	1.16 (0.91, 1.47)	0.225
Selenium (Se)	1.45 (0.57, 3.69)	0.423	1.01 (0.83, 1.22)	0.946	1.00 (0.80, 1.24)	0.975	1.03 (0.83, 1.27)	0.772	1.00 (0.80, 1.24)	0.969	0.99 (0.79, 1.24)	0.934	1.05 (0.83, 1.32)	0.69
Iron (Fe)	0.65 (0.26, 1.61)	0.346	**0.82 (0.68, 0.98)**	**0.028**	0.81 (0.64, 1.02)	0.074	0.84 (0.67, 1.06)	0.137	0.83 (0.65, 1.07)	0.147	0.84 (0.65, 1.09)	0.181	0.90 (0.68, 1.18)	0.423
Chromium (Cr)	0.57 (0.16, 2.10)	0.395	0.93 (0.71, 1.21)	0.579	0.83 (0.61, 1.14)	0.254	0.89 (0.65, 1.21)	0.45	0.88 (0.63, 1.21)	0.419	0.86 (0.62, 1.19)	0.351	0.98 (0.70, 1.37)	0.895

## Data Availability

The data presented in this study are openly available in Mendelei.

## Supplementary Materials

The following supporting information can be downloaded at: https://www.mdpi.com/article/10.3390/ijms241310968/s1.
