# Supplementary material for: The Impact of Essential Trace Elements on Ovarian Response and Reproductive Outcomes following Single Euploid Embryo Transfer
Source: Int J Mol Sci. 2023 Jun 30;24(13):10968. doi: 10.3390/ijms241310968 (PMC10341631; doi:10.3390/ijms241310968)
Supplement: Supplementary file 1 [file ijms-24-10968-s001.zip › ijms-2449623-supplementary.pdf]

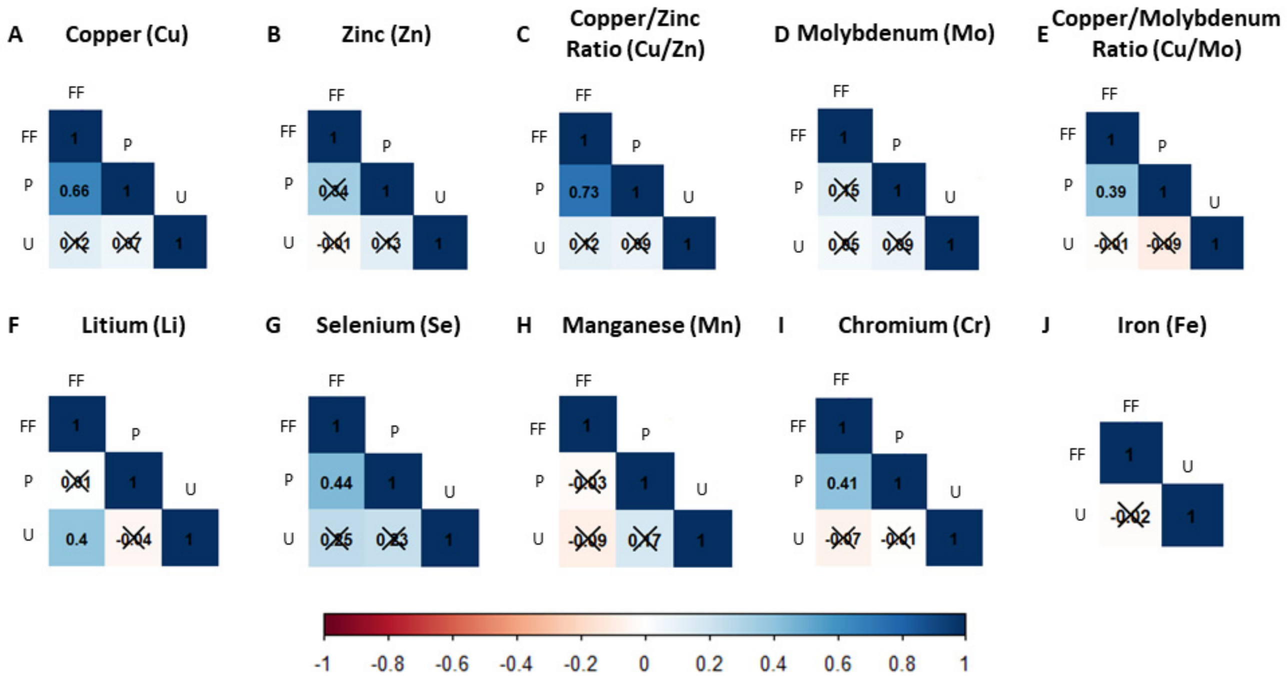

Figure S1. Spearman correlation matrix representing the relationships of each essential trace elements between biological matrices.

Blue represents a positive correlation, while red represents a negative correlation. Color intensity is proportional to the correlation strength. Strikethrough values represent insignificant/negligible associations (p>0.05). FF, follicular fluid; P, plasma; U, urine.

.

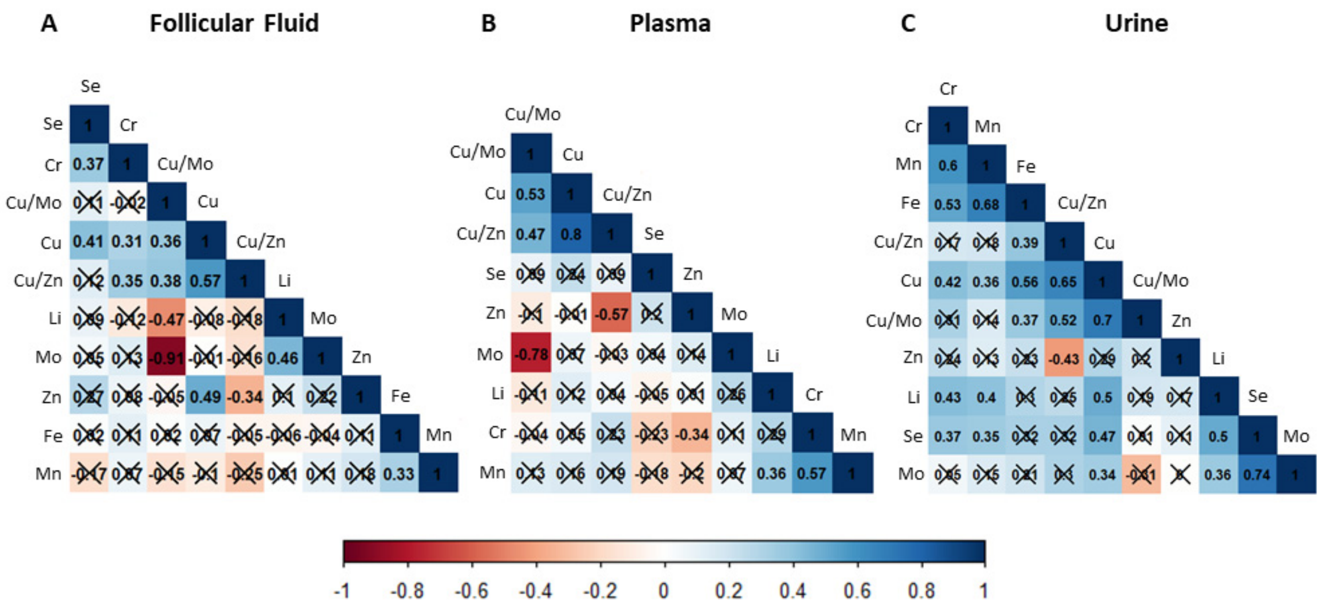

Figure S2. Spearman correlation matrix representing the relationships between the essential trace elements within each biological matrix.

Blue represents a positive correlation, while red represents a negative correlation. Color intensity is proportional to the correlation strength. Strikethrough values represent insignificant/negligible associations ($p>0.05$). Cr, chromium; Cu, copper; Fe, iron; Li, lithium; Mn, manganese; Mo, molybdenum; Se, selenium; Zn, zinc.
